# Modelling the phenology of the grapevine leafhopper *Erasmoneura vulnerata*

**DOI:** 10.1002/ps.71105

**Published:** 2026-07-10

**Authors:** Andrea De Francesco, Andrea Pintossi, Anna Simonetto, Igor Daniel Weber, Paola Gervasio, Gianni Gilioli

**Affiliations:** ^1^ Department of Civil, Environmental, Architectural Engineering and Mathematics University of Brescia Brescia Italy

**Keywords:** integrated pest management (IPM), Cicadellidae, vineyard, phenological model, stage‐structured model

## Abstract

**BACKGROUND:**

*Erasmoneura vulnerata* (Fitch) (Hemiptera: Cicadellidae) is an invasive Nearctic leafhopper that has become a pest of economic relevance in European vineyards. Despite its growing impact, published monitoring data remain scarce, and there is still a lack of tools to support decision‐making in the management of the pest. This study presents the first stage‐structured model describing its phenology and incorporating non‐linear temperature‐dependent development.

**RESULTS:**

The model was calibrated and validated leveraging field data collected in northern Italy during a continuous monitoring campaign conducted between March 2024 and July 2025, together with published monitoring data. Model parameters were estimated through an iterative optimization procedure that minimized the error between the simulated and observed population phenological progression of the pest during the calibration phase. Simulations showed a good fit between observed and simulated phenology across years and sites, with minor deviations possibly reflecting differences between local field conditions and the gridded temperature data used as input, as well as other sources of phenological variability not explicitly represented in the model.

**CONCLUSIONS:**

The proposed model provides a faithful and biologically grounded representation of *E. vulnerata* phenology under varying environmental conditions, offering a practical tool to assist decision‐making in the monitoring and control of the pest. © 2026 The Author(s). *Pest Management Science* published by John Wiley & Sons Ltd on behalf of Society of Chemical Industry.

## Graphical abstract

This study developed and validated a phenological population dynamics model for *Erasmoneura vulnerata*, integrating field monitoring and literature data to reproduce observed seasonal patterns across multiple sites.

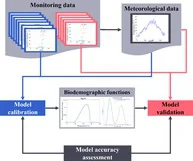

## 1 INTRODUCTION

The grapevine leafhopper *Erasmoneura vulnerata* (Fitch) (Hemiptera: Cicadellidae) is an invasive species originating from the Nearctic region (central and eastern USA, southern Canada, and northern Mexico).^1^ In 2004 it was first recorded outside of its native area of distribution in northern Italy^2^ and in the following years its presence was recorded in other European countries such as Slovenia,^3^ Romania,^4^ Switzerland,^5^ Serbia,^6^ Bulgaria,^7^ Hungary,^8^ and Moldova.^9^

The pest's primary hosts are wild and cultivated grapevines (*Vitis* spp.), but it feeds also on other plants such as *Parthenocissus quinquefolia*, *Parthenocissus tricuspidata*, *Rosa* spp., *Prunus* spp., *Cercis* spp., and *Ilex decidua* ^3^, ^10^, ^11^, ^12^ and it was recorded on more than 50 different species (for a complete list, see Dmitriev *et al*.^13^). Feeding occurs on the mesophyll cells of the leaves of the hosts, producing pale speckled areas that, at high population densities, coalesce and thereby reduce the photosynthetic capacity of the plant,^14^ ultimately anticipating leaf drop. In Europe, initial investigations suggested that the leafhopper would not represent a major threat in vineyards, as its population densities were expected to remain low due to the mandatory control measures already in place against *Scaphoideus titanus*.^2^ However, severe outbreaks with injuries affecting more than 90% of the canopy were recently reported,^15^ raising concerns among farmers. The probable cause of such outbreaks could be linked to *E. vulnerata* resistance to insecticides widely used in vineyards for controlling other leafhoppers.^16^

Within its native range, Zimmerman *et al*.^14^ reported that *E. vulnerata* develops through two generations on grapes in Colorado, whereas in northern Italy a third generation may be detected according to Duso *et al*.^11^ Adults overwinter outside vineyards in reproductive diapause, seeking shelter under loose bark, in evergreen plants, or under leaf litter,^2^, ^10^ but they can remain active and feed on alternate hosts whenever meteorological conditions are favorable. In spring, adults migrate back to vineyards at the onset of grapevine vegetative growth and oviposit in the vascular bundles of young leaves.^14^ Nymphs of the first generation appear from the second half of May and develop through five instars before emerging as adults. The abundance of this generation is usually low and does not cause significant damage, although feeding on new shoots in early spring may hinder vegetative growth. The second generation is considered the most harmful, as high population densities build up in mid‐summer.^10^, ^11^ A third generation may develop between August and September, although its peak abundance is generally lower than that of the second generation. From late summer to early autumn, adults enter reproductive diapause and migrate to overwintering sites. The generations are highly overlapped, as observed in other leafhopper species,^17^ with individuals of the same stage from different generations often occurring simultaneously, which makes it difficult to clearly distinguish one generation from another.

The growing impact of the pest in commercial vineyards, combined with its presence throughout the entire grapevine growing season, highlights the need for tools that can support decision‐making in pest management. Such tools should help identify the most suitable time windows for monitoring and control activities. Monitoring efforts should concentrate either on periods when the host is most susceptible (i.e. at the beginning of the vegetative season) or when the pest reaches its highest population density (i.e. during the second generation in northern Italy, according to Duso *et al*.^11^), when its impact is likely greatest. These windows could be determined by implementing a model capable of describing the phenology of the pest over time. However, no such model has yet been developed, and the only estimates of its thermal requirements are provided in Jarrell,^18^ who computed cumulative Degree Days using a lower developmental threshold of 10 °C.

In this work, a phenological model for *E. vulnerata* was developed to describe the seasonal progression of its life stages. The model adopts a stage‐structured approach in which development is driven by non‐linear temperature‐dependent rate functions. In addition, egg‐laying is described through two distinct fecundity functions depending on adult physiological age, allowing differences between overwintered and summer‐emerging adults to be explicitly represented. Model parameters were estimated directly from field and published monitoring data through an iterative calibration procedure, in the absence of controlled laboratory measurements. Model performance was then evaluated by comparing simulated and observed patterns of stage emergence.

## 2 MATERIALS AND METHODS

### 2.1 Data collection

Population dynamics data for both nymphs and adults were collected during a monitoring campaign conducted from March 2024 to July 2025. The study area was located in the Franciacorta wine‐producing region (Province of Brescia, northern Italy), in which two sites located in Coccaglio and Erbusco, respectively, were selected based on reports of *E. vulnerata* outbreaks in the previous years.

During the campaign, adults were monitored using yellow sticky traps (Glutor Giallo, Biogard, Grassobbio, Italy) placed both within the vineyards and the surrounding vegetation in order to obtain a comprehensive picture of the pest's population dynamics that included the adult overwintering phase. Nymphs were monitored by randomly collecting 20 grapevine leaves from each vineyard. Adults were counted with an automatic detection algorithm based on the YOLO (You Only Look Once) model (version 8s)^19^ specifically trained to recognise *E. vulnerata* adults from high‐resolution images. Automated counts were quality‐checked against manual counts on a subset of 281 trap images. Overall, the algorithm underestimated adult numbers by 9% relative to manual counts (mean difference = −41.7 individuals per trap; *t*‐test *P* = 0.0001). This level of underestimation was considered acceptable, also considering the possible human error inherent to manual counting. Nymphs, instead, were manually counted under a stereomicroscope (ZEISS Stemi 305, 5× magnification; Carl Zeiss microscopy GmbH, Jena, Germany). After counting, trap and nymph data referring to the same vineyard (or to the overwintering sites in the case of adults) were averaged to obtain a single representative time series for each vineyard or overwintering site.

In 2024, traps were installed in five vineyards (two in Coccaglio, covering a total of 1.2 ha, and three in Erbusco, covering a total of 3.4 ha) and in the surrounding overwintering sites in late March. Traps were arranged in vineyards following a regular grid of 20 × 20 m, corresponding to 25traps ha^−1^. In overwintering sites, a total of 37 traps were installed (nine in Coccaglio and 28 in Erbusco), positioned near secondary host plants with a minimum spacing of approximately 20 m where possible. They were replaced weekly until late November, after which vineyard monitoring was interrupted due to the absence of the pest, while monitoring in the overwintering sites continued throughout the winter and ended in July 2025. Between November 2024 and March 2025, traps located in the overwintering sites were substituted bi‐weekly due to the low abundance of captures. Registered captures were then divided by two to ensure comparability with the other capture data. Nymphs, instead, were monitored only within vineyards, starting from late May 2024 (corresponding to the first occurrence of nymphs in the vineyards) until early November 2024, after which no leaves remained on the vines.

In 2025, the number of monitored vineyards was reduced to two (one in Coccaglio, covering 0.8 ha, and one in Erbusco, covering 2.0 ha). In the other vineyards, control methods specifically targeting *E. vulnerata* populations were applied, which prevented a coherent analysis of the monitoring data. In the monitored vineyards, traps were placed in early March 2025 following a regular grid of 40 × 40 m, corresponding to 6.25 traps ha^−1^. A reduced trap density was adopted in 2025, as the coarser grid was sufficient to capture the temporal dynamics required for phenological modelling. In overwintering sites, traps were positioned following the same criteria adopted in 2024. Traps were replaced weekly until the end of the campaign in July 2025. Nymph monitoring, instead, began at the first occurrence of the stage in late May, as in 2024, and continued until the end of the monitoring campaign.

### 2.2 Dataset for model calibration and validation

The data collection procedure resulted in seven population dynamics series referred to vineyards (five series for 2024, plus two series for 2025). To these, six other series published in Duso *et al*.^11^ were added as a complement to the data collected in 2024 and 2025 to broaden the geographical and temporal scope of the model calibration and validation, resulting in a dataset comprising a total of 13 series (see Table 1). Population dynamics data from the USA^18^ were not included because differences in the genetic background of North American populations compared with those established in Italy could lead to distinct biodemographic responses, such as development and fecundity rates, potentially biasing the calibration of the model parameters.

**Table 1:** Series used for the model calibration and validation phases, with name of the series, location, year, and source of the series

Series name	Location	Year	Source	Calibration or Validation
A1_17	Alonte (VI)	2017	Duso *et al*.^11^	Calibration
A2_17	Alonte (VI)	2017	Duso *et al*.^11^	Validation
C1_24	Coccaglio (BS)	2024	This study	Validation
C2_24	Coccaglio (BS)	2024	This study	Validation
C1_25	Coccaglio (BS)	2025	This study	Calibration
E1_24	Erbusco (BS)	2024	This study	Calibration
E2_24	Erbusco (BS)	2024	This study	Calibration
E3_24	Erbusco (BS)	2024	This study	Calibration
E3_25	Erbusco (BS)	2025	This study	Validation
L1_18	Lonigo (VI)	2018	Duso *et al*.^11^	Calibration
L2_19	Lonigo (VI)	2019	Duso *et al*.^11^	Calibration
M1_18	Monteforte (VR)	2018	Duso *et al*.^11^	Calibration
M1_19	Monteforte (VR)	2019	Duso *et al*.^11^	Validation

Data on population dynamics obtained from both the monitoring campaign and the literature were first analysed to identify successive generations based on peaks in population density, while local minima were used to approximate the boundaries between generations. When generations overlapped, individuals were proportionally allocated to adjacent generations considering both the relative magnitude of the peaks and their temporal distance from the overlap boundaries. The data were then cumulated and normalized to 100, yielding cumulative values for each monitored stage in each generation. Then, each series was filtered to exclude from the calibration and validation data the cumulative captures of those stages for which monitoring began too late or ended too early, resulting in incomplete cumulative capture data. Therefore, the filtering procedure excluded (i) overwintering adults that were not monitored in their overwintering sites during winter and (ii) adults of the last generation.

Because overwintered adults resume activity in overwintering habitats before migrating to vineyards at budbreak, captures recorded only inside vineyards would mainly reflect the timing of vineyard colonization rather than the transition towards reproductive activity. For this reason, captures from overwintering sites were integrated to reconstruct the early phase of the overwintered adult phenology. For the C1_25 and E3_25 series (the only series for which overwintering adult captures could be assumed to be complete, owing to continuous trap monitoring during the 2024–2025 winter), the cumulative captures of the overwintered adult generation inside the vineyard (assumed here to serve as a proxy for the transition between adults resuming their activity after winter but still not in their reproductive phase, and mature adults capable of reproducing) were corrected by integrating the capture data from the overwintering sites. Specifically, captures from overwintering sites were rescaled to make them comparable with those from vineyards by multiplying their original values by the ratio between the peak captures recorded in vineyards and those in overwintering sites. The rescaled overwintering‐site captures were then added to the vineyard series starting from the first date when a clear increase in captures was observed in the overwintering sites (around early February) and ending on the first date when such an increase was recorded in the vineyards (in the second half of March). This procedure allowed obtaining a more faithful representation of the phenology of overwintered adults, which would otherwise lack information on the resumption of activity prior to migration towards vineyards at budbreak.

After the computation of the cumulative normalized data, the 13 series were subdivided between the calibration and validation datasets, with eight series dedicated to the calibration phase and the remaining five series to the validation phase (see Table 1, which summarizes the allocation of all series to the calibration and validation datasets, including series from both the 2024–2025 monitoring campaigns and series obtained from literature). Series were subdivided between the two phases ensuring equal representation of different years and locations in order to minimize overfitting and test the model against independent conditions.

### 2.3 Temperature data

Temperature data were obtained from the ERA5‐Land dataset,^20^, ^21^ provided by the Copernicus Climate Change Service (C3S) through the Climate Data Store (CDS), which offers hourly meteorological variables data at a 0.1° × 0.1° spatial resolution. Hourly data (air temperature at 2 m from the ground) were downloaded for each of the combinations of location and year by selecting the coordinates of a bounding box encompassing the location of the monitored fields.

### 2.4 Daylength data

Daylength data were computed for each of the monitoring locations with the daylength function included in the geosphere R package,^22^ requiring as inputs only the latitude and the range of days of the year for which daylength values are to be computed (in this case, 1:365).

### 2.5 Model description

The phenology of *E. vulnerata* is simulated with a stage‐structured population model based on Kolmogorov's partial differential equation system (see Gilioli *et al*.^23^, ^24^ for examples of the application of the modelling framework and Gilioli *et al*.^25^ for a more thorough description of the model's derivation), which considers two different dimensions, namely time *t* and physiological age *x*. For each stage i, let $\varphi_{i}\left(t,x\right)\mathrm{dx}$ be the number of individuals at time *t* with physiological age between x and x+dx, with $x\in \left[0,1\right]$. The dynamics of each stage are described by:

$$\frac{\partial \varphi_{i}\left(t,x\right)}{\partial t}+\frac{\partial }{\partial x}\left[d_{i}\left(T\left(t\right)\right)\varphi_{i}\left(t,x\right)-\sigma_{i}\frac{\partial \varphi_{i}\left(t,x\right)}{\partial x}\right]=0,\;t>0,x\in \left(0,1\right)$$

where $d_{i}\left(T\left(t\right)\right)$ is the stage‐specific development rate function and $\sigma_{i}$ is the diffusion coefficient, accounting for variability in development among individuals within the same stage. In the model, individuals enter each stage through an incoming flux at the beginning of the physiological‐age domain and leave the stage when they complete their physiological development. For stages other than eggs and the initial overwintering adult stage A0, the incoming flux is determined by the outflow from the previous stage. For eggs, the incoming flux is generated by the age‐dependent fecundity of reproductive adults, either A1 or A2 depending on the generation considered. The cumulative values of the stage‐specific incoming fluxes represent the cumulative emergences predicted by the model. These cumulative emergences are the quantities used for calibration and validation against the normalized cumulative monitoring data. The *E. vulnerata* populations are structured in five different stages, and the transitions between subsequent stages are dependent on development and fecundity rate functions (Fig. 1).

**Figure 1:**
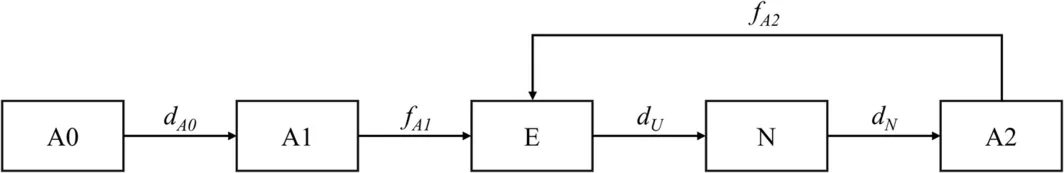
Schematic representation of the model structure. The stages in which the life cycle of the pest has been subdivided are shown in the rectangles. A0 are the overwintered adults before the reproductive phase; A1 are overwintered adults in the reproductive phase; E are eggs; N are nymphs; A2 are adults that did not overwinter. Arrows represent processes (development and fecundity) governing the transitions between stages. Each stage has its own development function (*d*_*i*_), and the reproductive adult stages (A1 and A2) are characterized by a fecundity function (*f*_*i*_).

At the beginning of the cycle, the overwintering adult stage has been divided in two substages (A0 and A1), representing the overwintering adult that is still in reproductive diapause (A0) and the overwintered adult in its reproductive phase (A1). A0 adults are initialized assuming they are distributed in their physiological age following a Beta distribution, with α=5 and β=2, representing an overwintering population that has already completed part of its development before the start of the simulation. Once daylength exceeds a threshold of 9.8 h, determined from observations on the resumption of adult activity within overwintering sites during winter 2025, overwintering adults in diapause (A0) begin their development. On completion of this stage, they become reproductive adults (A1). This threshold therefore controls the onset of post‐winter development. Following the A1 adults are the egg (E), nymphs (N, considered as a single stage and not divided into substages), and the non‐overwintered adults (A2). Each stage is characterized by its own development rate function, describing the rate at which each stage progresses through its physiological age, depending on ambient temperature. The development rate functions of each stage are modelled with the Briere function^26^:

$$d_{i}\left(T\left(t\right)\right)=\left\{\begin{array}{l} \frac{k_{i}T\left(t\right)\left(T\left(t\right)-\mathrm{Tin}f_{i}\right)\sqrt{\mathrm{Tsu}p_{i}-T\left(t\right)}}{24},\\ \;\mathrm{Tin}f_{i}\leq T\left(t\right)\leq \mathrm{Tsu}p_{i}\\ 0,\\ \;\;T\left(t\right)<\mathrm{Tin}f_{i}\vee T\left(t\right)>\mathrm{Tsu}p_{i} \end{array}\right.$$

where $k_{i}$ is an empirical constant depending on stage i (where i=1,…,5), $T\left(t\right)$ is the hourly air temperature, and $\mathrm{Tin}f_{i}$ and $\mathrm{Tsu}p_{i}$ are the lower and upper development temperature thresholds of the ith stage, respectively.

The fecundity rate functions describe the physiological age‐dependent oviposition pattern of the reproductive adults (A1 and A2): for each increase in the physiological age of the adult, a fraction of an egg is laid, totaling a whole egg at the completion of the physiological age of the adult. The fecundity rate functions are modelled using a Beta distribution:

$$f_{i}\left(x_{i}\left(t\right)\right)=\frac{x_{i}{\left(t\right)}^{\alpha_{i}-1}{\left(1-x_{i}\left(t\right)\right)}^{\beta_{i}-1}\Gamma \left(\alpha_{i}+\beta_{i}\right)}{\Gamma \left(\alpha_{i}\right)\Gamma \left(\beta_{i}\right)}$$

where $x_{i}$ is the physiological age of the ith stage (i=2,5), and $\alpha_{i}$ and $\beta_{i}$ are the shape parameters of the Beta distribution. The choice of using a Beta distribution to model the fecundity as a function of the adult's physiological age allows the model to be conservative, ensuring that each adult produces exactly one egg by the end of its development. At each timestep of the simulation, each adult in its reproductive phase progresses in its physiological age and generates a fraction of an egg. The sum of all egg fractions laid by an adult equals one, representing one egg. All adults of each generation contribute to the number of eggs laid in a timestep.

Stage‐specific mortality was not included in the present model, as the focus of this study is on temperature‐dependent development and fecundity, which primarily determine the timing and succession of developmental stages, while mortality primarily affects abundance rather than the timing of stage progression.

### 2.6 Calibration and validation procedure

The series included in the calibration dataset were used to calibrate the model via an iterative procedure written in Matlab (ver R2023a).^27^ The procedure was based on a combination of the particleswarm and patternsearch algorithms (two derivative‐free optimization methods, which are part of the Matlab Global Optimization Toolbox) on the calibration datasets, aimed at minimizing the error between the cumulates of the monitoring data for each stage within each generation and the corresponding cumulates of the emergences simulated by the model. The particleswarm optimization algorithm searches for optimal parameter combinations by simulating the coordinated movement of a group of particles through the parameter space. Each particle represents a candidate solution and updates its position according to both its own best experience and that of the swarm, progressively converging toward promising regions of the search space.^28^ The best solution identified by this global search is then passed to patternsearch, a derivative‐free local optimizer that systematically explores neighboring points to refine the minimum with higher precision.^29^ This combined approach leverages the advantages of both algorithms, allowing particleswarm to first explore the parameter space in order to roughly identify the position of the global minimum, while the subsequent patternsearch algorithm refines the global minimum position.^30^ To implement this procedure, the lower and upper boundaries of each parameter defining the development rate and fecundity functions were defined (see Table 2). The boundaries were chosen based on available information in the literature regarding *E. vulnerata* ^19^ and other leafhoppers^31^, ^32^, ^33^ and were intentionally set broad enough to allow the particleswarm algorithm to explore a parameter space likely encompassing the true parameter values, as no experimental data were available to directly parameterize temperature‐dependent development and fecundity functions for *E. vulnerata*. Upper bounds were not further extended, even when reached during calibration, to avoid biologically unrealistic parameter combinations and to prevent the exploration of temperature ranges not supported by the observed environmental conditions.

**Table 2:** Lower and upper boundaries for each parameter of the development and fecundity functions for all stages during the model calibration phase

Stage	Function	Parameter	Lower boundary	Upper boundary
A0	Development	*k*_*A0*_	0.00001	0.0001
		*Tinf*_*A0*_	5	15
		*Tsup*_*A0*_	25	40
A1	Development	*k*_*A1*_	0.00001	0.0001
		*Tinf*_*A1*_	5	15
		*Tsup*_*A1*_	25	40
	Fecundity	*α*_*A1*_	1.01	60
		*β*_*A1*_	5	30
E	Development	*k*_*E*_	0.00001	0.0004
		*Tinf*_*E*_	5	15
		*Tsup*_*E*_	25	40
N	Development	*k*_*N*_	0.00001	0.0001
		*Tinf*_*N*_	5	10
		*Tsup*_*N*_	25	40
A2	Development	*k*_*A2*_	0.00001	0.0001
		*Tinf*_*A2*_	5	15
		*Tsup*_*A2*_	25	40
	Fecundity	*α*_*A2*_	1.01	60
		*β*_*A2*_	5	30

The particleswarm algorithm was configured with a swarm size of 100, a maximum of 300 iterations, and a function tolerance of 10^−4^. The patternsearch step was executed in parallel mode with function and step tolerances of 10^−4^ and 10^−6^, respectively.

After the minimization procedure reached convergence to a set of parameters for the development rate functions and fecundity rate functions, the obtained values were used to test the model on the validation dataset. For each series in both the calibration and the validation datasets, the mean absolute error (MAE) was computed in R (version 4.2.1)^34^ as a quantitative measure of the accuracy of the model. The MAE was calculated in two ways: (i) by comparing the cumulative abundances simulated by the model with those observed in the monitoring datasets and (ii) by comparing the time at which corresponding cumulative values were reached in the simulations and the observations. This procedure produced accuracy estimates for each series used in both calibration and validation, allowing the accuracy of the model for each series to be evaluated separately. Moreover, to obtain a more comprehensive view of the overall accuracy of the model, the MAE was then averaged across series, separately for the calibration and validation datasets. This averaging approach allowed each series to be given equal weight, regardless of differences in the number of stages or generations represented, or in the number of monitoring data points within each series.

## 3 RESULTS

### 3.1 Model calibration

From the calibration procedure, parameter values for both the development rate functions and the fecundity rate functions were obtained. The temperature‐dependent development rate functions for each of the modelled stages are represented in Fig. 2. The estimated lower and upper temperature thresholds differed among stages, encompassing the whole of the temperature ranges defined by the boundaries that were set for the minimization procedure. Both nymphs and A1 adults reached the upper boundary in both Tinf and Tsup, while A0 adults reached the lower boundary for Tsup, and A2 adults reached the lower boundary for Tinf. Peak development rates were between 20 and 25 °C for eggs and A0 adults, while for nymphs, A1 and A2 adults they were between 25 and 35 °C.

**Figure 2:**
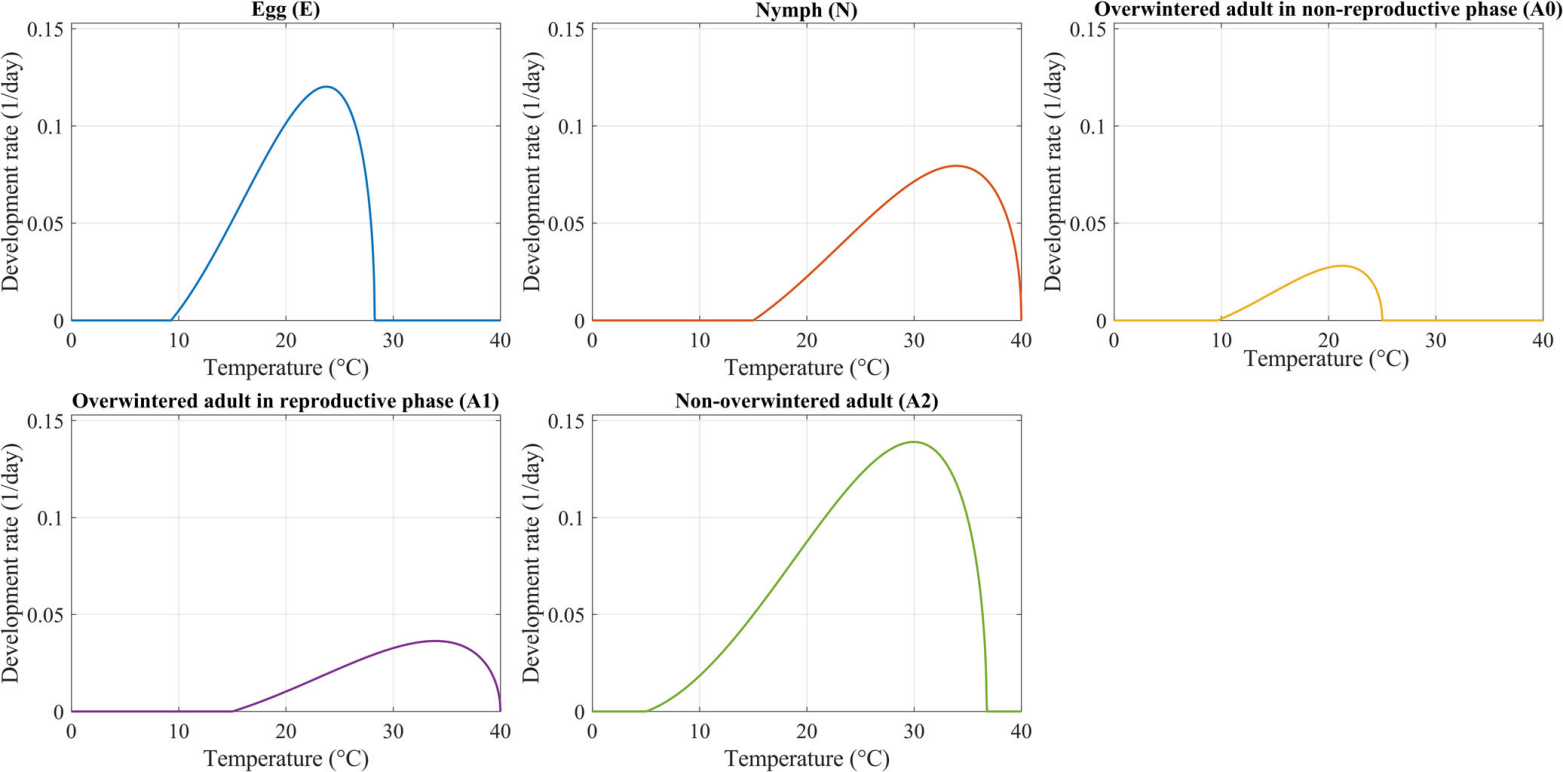
Development rate functions for each of the stages in which the life cycle of the pest has been structured in the model.

In Fig. 3, the two physiological age‐dependent fecundity rate functions are shown: one for A1 (the overwintered adult that resumed its reproductive activity) and one for A2 (the adult stage emerging during the grapevine growing season). Notably, oviposition occurs earlier in the physiological age of A1 adults than in that of A2 adults and it spans a narrower range of physiological age. Peak fecundity occurs around a physiological age of 0.2 for the A1 adult, while that of A2 occurs between 0.6 and 0.7.

**Figure 3:**
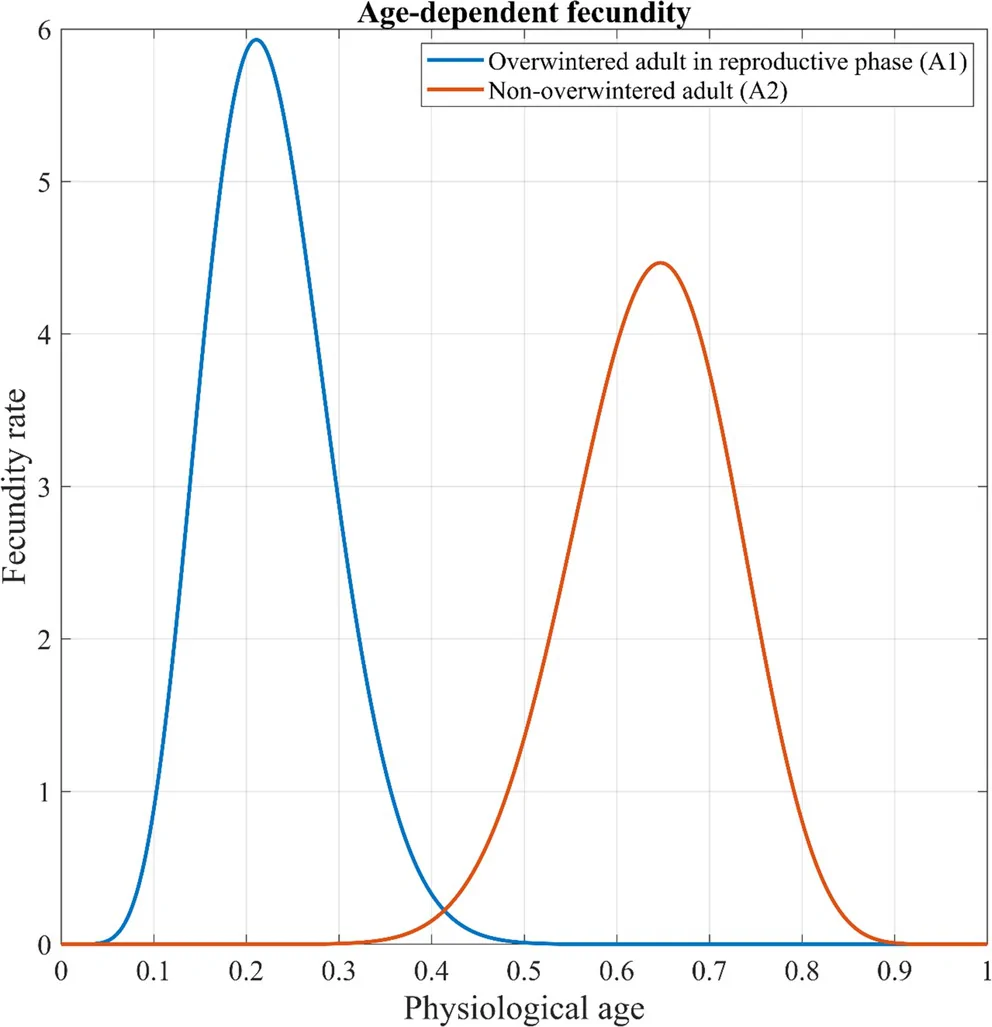
Fecundity functions for the two adult reproductive stages: overwintered adults in their reproductive phase (A1) and non‐overwintered adults (A2).

As described in the Materials and Methods section, the accuracy of the model for the calibration phase was computed by comparing both the cumulative values simulated by the model with those observed in the monitoring, and the times at which corresponding cumulative values were reached in the simulations and in the observations. Values of the accuracy metric for each series used in the calibration procedure, together with their average, are presented in Table 3. Average MAE computed by comparing cumulative values was 3.31, indicating an average model error of slightly more than 3% between observed and simulated cumulative emergences, while the same metric computed by comparing the time at which corresponding cumulative values are reached in the field respect to the model predictions was 5.68 days, indicating an average absolute error of less than 6 days. Looking at the metric computed for each series separately, the model performed best in representing the phenological pattern of the L1_18 (best MAE‐time) and M1_18 (best MAE‐value) series, while the worst performance was obtained for the L2_19 series.

**Table 3:** Accuracy for the calibration phase.

Series name	MAE time	MAE value
A1_17	4.556122	3.404698
C1_25	7.067883	4.178434
E1_24	5.38382	2.567567
E2_24	5.537341	3.031514
E3_24	7.830558	3.493602
L1_18	3.546138	2.680512
L2_19	7.858173	4.770456
M1_18	3.66387	2.335659
**Average**	**5.680488**	**3.307805**

The mean absolute error (MAE) was computed by comparing the dates at which corresponding cumulative values were reached in the simulations and the observations (MAE time), and by comparing the cumulative abundances simulated by the model with those observed in the monitoring datasets (MAE value).

Looking at the plots representing the cumulative emergences resulting from model calibration, compared with the field‐collected data (Fig. 4), the model predictions align well with the capture data, with few notable deviations. Regarding the first generation of nymphs (orange asterisks representing field data and orange lines representing model predictions), in the C1_25 series the model tends to lag behind the monitoring data by an average of 8.4 days, whereas for L2_19 it predicts earlier emergences by an average of 7.8 days. The first generation of adults (yellow asterisks and lines for field data and model predictions, respectively) is well described by the model in four out of seven series in which data for this stage and generation were collected, with only a minor anticipation of the predictions relative to the field data for the A1_17 series (average error of 5.0 days). However, in the series located in Erbusco, the model shows a delay at the onset of emergence in the E1_24 and E2_24 series (average of 10.6 and 8.5 days, respectively) and an evident anticipation of the cumulative emergence pattern from approximately the first fifth of the cumulative curve onwards in all three Erbusco series (average of 7.3 days for E1_24 and E2_24 and of 11.3 days for E3_24). A similar pattern can be seen for the second generation of nymphs (purple asterisks and lines), but in this case the model predictions always anticipate the field data in the Erbusco series (with an average error between 8.2 and 10.3 days). The following stages (second generation of adults in green and third generation of nymphs in sky blue), are generally well‐fitted in all the series used for the calibration procedure, with the only exception of the series L2_19, where the model lags behind the field data (average error of 14.3 days for the second generation of adults and 8.3 days for the third generation of nymphs). In A1_17, instead, a slight anticipation of the model predictions with respect to the field data can be observed, with an average anticipation of 3.8 days for the second generation of adults and an average anticipation of 6.6 days for the third generation of nymphs.

**Figure 4:**
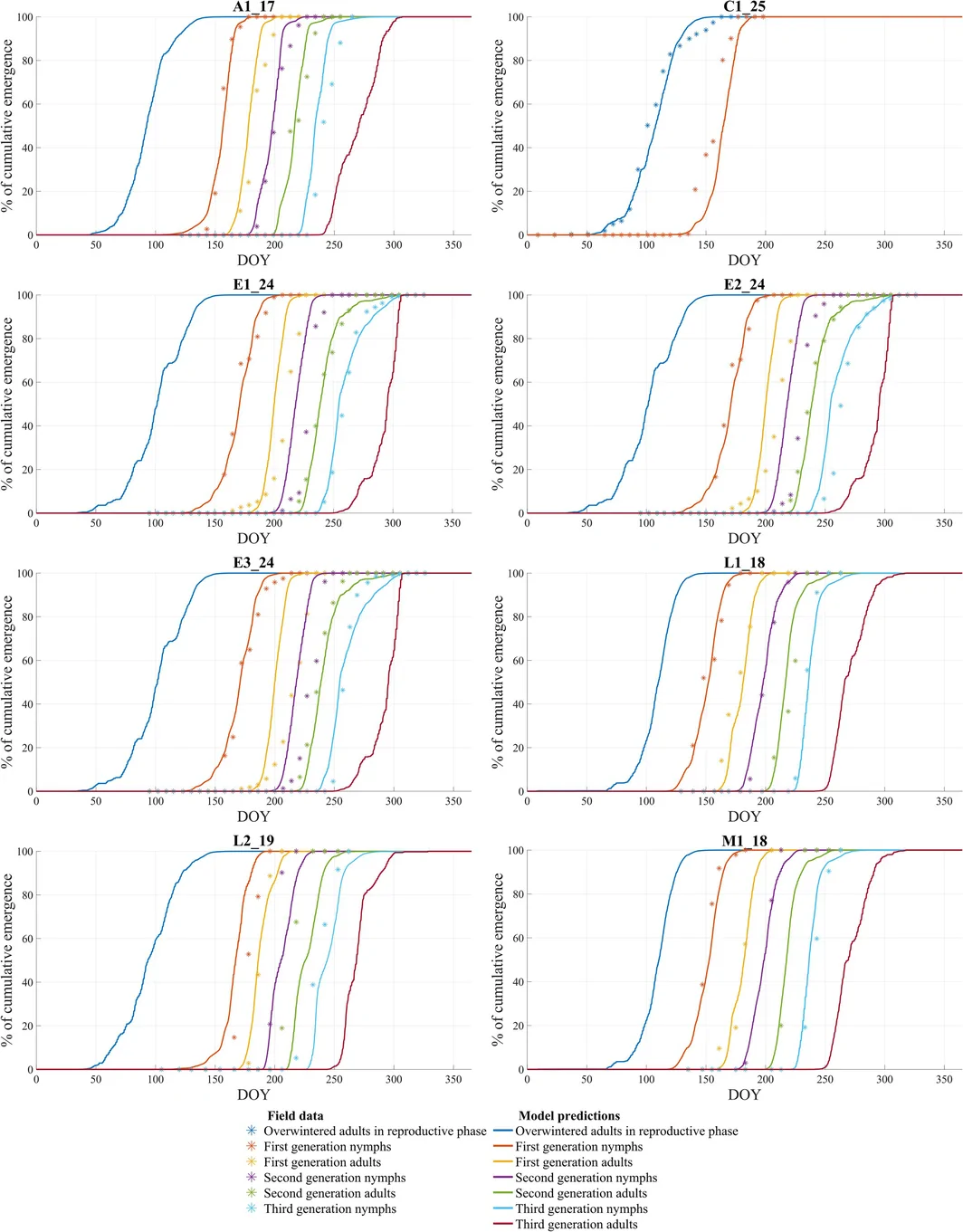
Results of the calibration phase. The graphs represent the sampled (asterisks) and estimated (lines) cumulative emergences (%) of *Erasmoneura vulnerata* stages for the series used during calibration. The *x* axis indicates the day of the year (DOY) and the titles of each graph correspond to the series name.

### 3.2 Model validation

The validation procedure, as described in the Materials and Methods section, was performed by implementing the model using five temperature series and by comparing the model predictions with the cumulative monitoring data. Table 4 presents the MAE computed for each series and its average. Average MAE slightly improved with respect to the calibration average, reaching respectively 5.13 days for the time‐based metric and 3.06 for the value‐based metric. The model performed best in representing the phenology of *E. vulnerata* in the C2_24 series, which reached the lowest values of both MAE‐time and MAE‐value, while the worst performance was recorded for the E3_25 series, which instead reached the highest value of MAE for both the assessed dimensions.

**Table 4:** Accuracy metrics for the validation phase.

Series name	MAE time	MAE value
A2_17	3.649156	2.672257
C1_24	6.869748	3.37386
C2_24	3.361865	1.436056
E3_25	7.863085	4.808208
M1_19	3.912694	3.028825
**Average**	**5.13131**	**3.063841**

The mean absolute error (MAE) was computed by comparing the dates at which corresponding cumulative values were reached in the simulations and the observations (MAE time), and by comparing the cumulative abundances simulated by the model with those observed in the monitoring datasets (MAE value).

A closer look at the plots representing the cumulative emergences resulting from the model validation and their corresponding field data (Fig. 5) reveals a very good alignment between observed and predicted values. A first notable exception concerns the emergence curve of the overwintered adults entering their reproductive phase (series E3_25, blue line and asterisks), for which the model anticipates the dynamics from approximately 85% of the cumulative curve onwards by an average of 12.5 days. In the same series, the first generation of nymphs (orange lines and asterisks) is delayed by the model relative to the field data by an average of 8.6 days, whereas in the M1_19 series it is slightly anticipated (average error is 6.4 days). The only other visible misalignment between model predictions and field data concerns the first generation of adults (yellow) in the C1_24 series, for which the model anticipates the observed trend by an average of 7.7 days. Overall, the remaining stages and generations are satisfactorily described by the model, showing a consistent agreement between predicted and observed cumulative trends.

**Figure 5:**
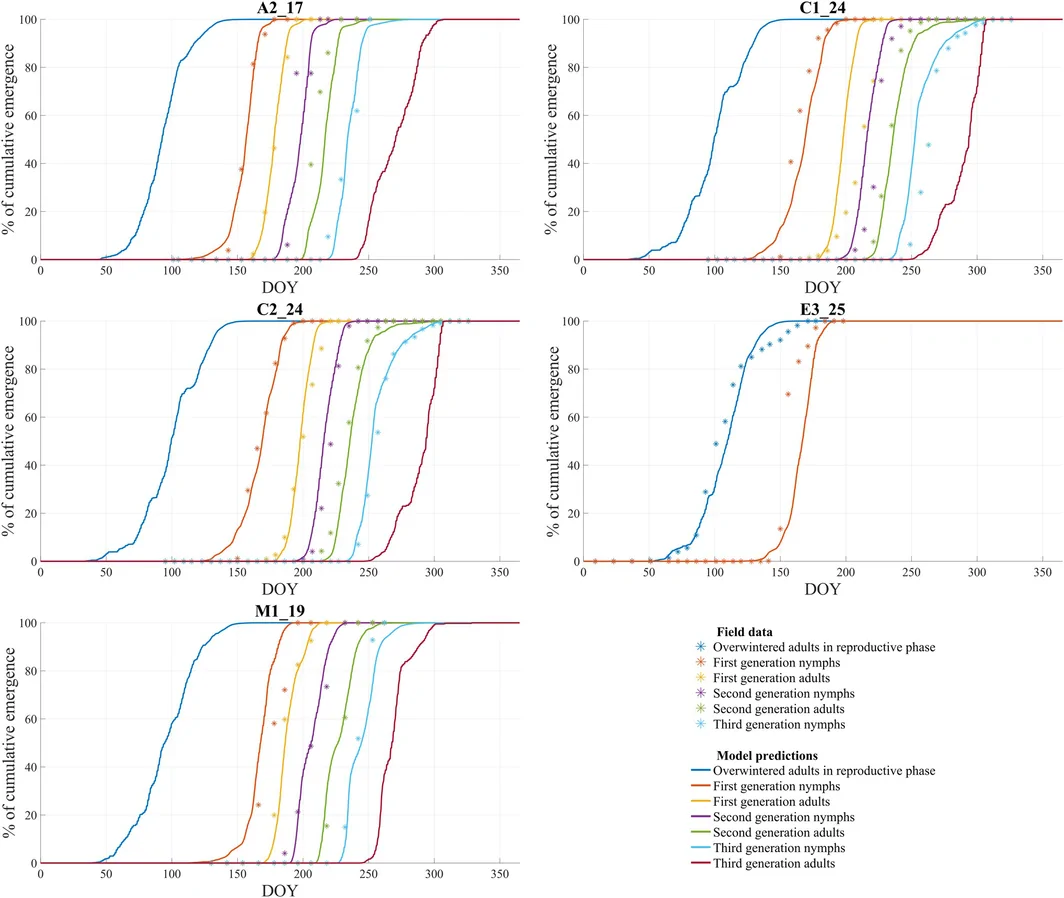
Results of the validation phase. The graphs represent the sampled (asterisks) and estimated (lines) cumulative emergences (%) of *Erasmoneura vulnerata* stages for the series used during validation. The *x* axis indicates the day of the year (DOY) and the titles of each graph correspond to the series name.

## 4 DISCUSSION

The calibrated functions describing temperature‐dependent development and age‐dependent fecundity produced biologically plausible responses and allowed the model to reproduce the phenology of *E. vulnerata* across sites and years. The calibration of all model parameters was required by the absence of experimental information on temperature‐dependent development and fecundity for *E. vulnerata* (direct experimental data under controlled thermal regimes would allow a more direct parameterization of these relationships). In such non‐linear multi‐parameter frameworks, the estimation problem is inherently ill‐posed, as different parameter combinations may lead to similar model outputs, while the limited number of field series further constrains parameter identifiability.^35^ To address this, a hybrid global–local optimization strategy was adopted to effectively explore the parameter space and identify solutions consistent with the observed phenology and with known life‐history traits and temperature‐dependent responses of the species and related taxa. Developmental thresholds were consistent with those reported for closely related Typhlocybinae leafhoppers, whose lower thresholds typically ranged between 7 and 16 °C and upper thresholds between 27 and 33 °C.^31^, ^32^, ^33^ While specific thermal limits for *E. vulnerata* have not been experimentally determined, the calibrated developmental functions behaved consistently with these general patterns, supporting their biological plausibility. The relative differences among stages also align with their seasonal occurrence: overwintered adults (A0) showed the lowest upper thresholds, consistent with their activity during the coldest part of the year, whereas nymphs and reproductive adults exhibited higher thresholds, reflecting their development during late spring and summer, when temperatures are typically highest. Such a pattern is expected under an adaptive interpretation of thermal development, whereby stages routinely exposed to cooler conditions tend to exhibit lower thermal limits, while stages developing under warmer conditions tend to show higher thresholds to optimize heat accumulation and ensure timely completion of development. This correspondence supports the internal coherence of the calibrated thermal responses.

Nymphal development times derived from the calibrated curve are broadly comparable with those reported in controlled‐environment experiments by Duso *et al*.,^10^ whereas egg development appears shorter than their inferred estimates. This discrepancy is difficult to interpret because egg duration in Duso *et al*.^10^ was estimated indirectly from nymph emergence rather than observed directly, and because our calibration lacked egg‐specific data. The minimization procedure may therefore have compensated by adjusting the timing of oviposition and egg development. By contrast, the two calibrated fecundity curves (Fig. 3) resulted in patterns that closely match the biology of related leafhopper species. Following the pre‐oviposition period represented in the model by the development of A0, overwintered adults exhibit a long reproductive period that may span several weeks.^36^, ^37^ In the model, this pattern emerges from the combination of low development rates during A1 and the age‐dependent distribution of fecundity. Non‐overwintered adults begin oviposition at a later physiological age (around 0.3–0.35; Fig. 3), but due to higher development rates at typical summer temperatures this corresponds to a short delay after adult emergence, typically within 3–7 days, consistent with observations reported for related leafhopper species.^36^, ^37^, ^38^ These consistencies provide confidence that the model realistically represents reproductive dynamics, despite the limited direct information available for *E. vulnerata*.

Model performance during calibration and validation was generally high, with average errors of approximately 3–4% in cumulative values and 5–6 days in timing. Deviations observed in some Erbusco series likely reflect differences between local field temperatures and ERA5‐Land data, whose 0.1° resolution may not capture fine‐scale thermal variability in areas with complex topography. Other sources of variability may also contribute, including natural variability in phenological responses and the influence of environmental drivers not explicitly included in the model. Since temperature directly governs development rates, even modest discrepancies can shift the simulated phenology relative to field observations, a pattern consistent across all Erbusco datasets. A lower fit for the L2_19 series is also plausible, as this vineyard received pyrethrin treatments during the season. Given that the efficacy of pyrethrins against *E. vulnerata* has been documented,^16^ such applications may have reduced adult or nymph abundance independently of phenological timing, thereby altering cumulative capture curves and reducing the apparent model fit.

Beyond its biological consistency, the main value of the model lies in its ability to provide stage‐specific and time‐resolved predictions of *E. vulnerata* phenology. These outputs can support vineyard management by indicating when each developmental stage is expected to occur, helping to organize monitoring activities and interpret trap captures in relation to the underlying phenological progression. In practical terms, the model can assist growers and advisors in identifying the most appropriate time windows for monitoring and control interventions targeting the most susceptible life stages. This information is particularly useful for planning monitoring during the second generation, which represents the period when *E. vulnerata* is most relevant from a management perspective in northern Italy.^16^ By offering a clear temporal framework for the succession of stages, the model helps align field observations with the expected timing of development, facilitating more informed and timely decisions. The model can also support the design of more sustainable control strategies by indicating when sensitive stages are expected to occur, allowing treatments to be better synchronized with pest development. This can help avoid unnecessary or poorly timed insecticide applications, which is particularly important given the susceptibility of key natural enemies to commonly used products in vineyards as in the case of *Chrysoperla carnea* and *Orius majusculus* (two generalist predators known to attack *E. vulnerata*), which are often limited in vineyards because of their sensitivity to pesticides.^39^ Preserving this kind of predator may enhance their contribution to pest suppression, especially in organic or low‐input systems. Adding to these, egg parasitoids of the genus *Anagrus*, including *A. atomus*, have also been detected in association with *E. vulnerata* oviposition sites in northeastern Italian vineyards,^40^ suggesting a potential role in biological control. Because *Anagrus* spp. adults are readily captured on yellow sticky traps,^41^, ^42^ identifying periods of oviposition or early‐stage development can help avoid placing traps for monitoring or for mass‐trapping at times when parasitoid presence is expected to be highest. In this way, phenological information on the pest can contribute to limiting unintended interference with natural enemy activity, supporting more selective and ecologically compatible interventions. More generally, the availability of phenological forecasts can contribute to improving the timing and selectivity of pest management interventions within integrated pest management programmes.

## 5 CONCLUSIONS

The model developed in this study effectively reproduces the phenological pattern of *E. vulnerata* across different years and locations, providing a scientifically grounded tool that can be used to support decision making in an integrated pest management framework. By incorporating non‐linear relationships between development rates and temperature, the model can describe the pest's phenology in different climatic conditions, making it applicable in contexts where it is reasonable to assume that *E. vulnerata* populations share the same physiological responses to temperature as those observed in the locations used for the model calibration and validation. Future work could extend the geographical and temporal scope of the empirical datasets to refine the underlying development and fecundity functions and improve the model's generality.

## CONFLICTS OF INTEREST

The authors declare that they have no conflicts of interest.

## Data Availability

The data that support the findings of this study are available from the corresponding author upon reasonable request.
